# Alternations of neuromagnetic activity across neurocognitive core networks among benign childhood epilepsy with centrotemporal spikes: A multi-frequency MEG study

**DOI:** 10.3389/fnins.2023.1101127

**Published:** 2023-02-22

**Authors:** Siyi Wang, Yingfan Wang, Yihan Li, Jintao Sun, Pengfei Wang, Kai Niu, Yue Xu, Yanzhang Li, Fangling Sun, Qiqi Chen, Xiaoshan Wang

**Affiliations:** ^1^Department of Neurology, The Affiliated Brain Hospital of Nanjing Medical University, Nanjing, China; ^2^MEG Center, The Affiliated Brain Hospital of Nanjing Medical University, Nanjing, China

**Keywords:** benign childhood epilepsy with centrotemporal spikes, cognitive function, Magnetoencephalography, spectral power, multi-frequency

## Abstract

**Objective:**

We aimed to investigate the alternations of neuromagnetic activity across neurocognitive core networks among early untreated children having benign childhood epilepsy with centrotemporal spikes (BECTS).

**Methods:**

We recorded the Magnetoencephalography (MEG) resting-state data from 48 untreated children having BECTS and 24 healthy children. The fourth edition of the Wechsler Intelligence Scale for Children (WISC-IV) was utilized to divide the children with BECTS into two groups: the cognitive impairment (CI) group with a full-scale intelligence quotient (FSIQ) of < 90 and the cognitive non-impairment (CNI) group with an FSIQ of > 90. We selected 26 bilateral cognitive-related regions of interest based on the triple network model. The neurocognitive core network spectral power was estimated using a minimum norm estimate (MNE).

**Results:**

In the CNI group, the spectral power inside the bilateral anterior cingulate cortex (ACC) and the bilateral caudal middle frontal cortex (CMF) enhanced within the delta band and reduced within the alpha band. Both the CI and the CNI group demonstrated enhanced spectral power inside the bilateral posterior cingulate cortex (PCC), bilateral precuneus (PCu) region, bilateral superior and middle temporal cortex, bilateral inferior parietal lobe (IPL), and bilateral supramarginal cortex (SM) region in the delta band. Moreover, there was decreased spectral power in the alpha band. In addition, there were consistent changes in the high-frequency spectrum (> 90 Hz). The spectral power density within the insula cortex (IC), superior temporal cortex (ST), middle temporal cortex (MT), and parahippocampal cortex (PaH) also decreased. Therefore, studying high-frequency activity could lead to a new understanding of the pathogenesis of BECTS.

**Conclusion:**

The alternations of spectral power among neurocognitive core networks could account for CI among early untreated children having BECTS. The dynamic properties of spectral power in different frequency bands could behave as biomarkers for diagnosing new BECTS.

## 1. Introduction

Benign epilepsy with centrotemporal spikes (BECTS) or rolandic epilepsy has been the most common form of childhood idiopathic focal epilepsy. It is estimated to account for 8–25% of all childhood epilepsies (Shinnar et al., 1999). It is placed among idiopathic localization-related epilepsy due to its unique onset age, seizure characteristics, neuroimaging, and electroencephalographic features (Gkampeta and Pavlou, 2012).

The onset age for BECTS is typically 4–16 years, with a peak incidence between 7–9 years, and seizures usually recede before age 16 (Bouma et al., 1997), called self-relief. More than 90% of children undergo epilepsy activation for about 3 years (Bouma et al., 1997). In reality, the phenomenon of experiencing fewer than five seizures occurs in 90% of children (Loiseau and Beaussart, 1973; Lerman and Kivity, 1975). Seizures are closely associated with sleep, often during drowsiness or wakefulness (Wirrell, 1998). Typical electroencephalography (EEG) indicates normal background with interictal epileptic discharges situated in the centrotemporal areas. Drowsiness and sleep elevate the discharge rate. Therefore, it is common for children with rare seizures to have abundant EEG activity (Fejerman, 2009).

The International Classification of Epilepsies and Epileptic Syndromes has defined BECTS as a Syndrome characterized by simple partial, hemifacial motor seizures involving the oropharyngeal muscles and tend to develop into generalized tonic-clonic seizures (No authors listed, 1989). BECTS is considered to have a relatively benign course traditionally, however, there is abundant evidence for neuropsychological impairment now (Baglietto et al., 2001; Nicolai et al., 2006; Danielsson and Petermann, 2009; Tedrus et al., 2009; Jurkevičienė et al., 2012; Verrotti et al., 2014), such as cognitive deficits, academic problems, attention deficits, language impairment, emotional disorders, and behavioral disturbance. In a study of 20 children with BECTS, Staden et al. (1998) found that 13 had language disabilities, including reading, spelling, and auditory language learning disabilities. Genizi et al. (2012) reported that children diagnosed with BECTS demonstrated significant impairment in “affective Theory of Mind” tasks. Furthermore, researchers found that 61 BECTS patients from another study displayed higher scores for sleep and behavioral problems (anxiety/depression, social issues, and aggressive behavior) than the healthy controls (Samaitienė et al., 2013).

The mechanism of cognitive impairment (CI) in patients with BECTS is complex and unclear. Frequent epileptic discharges may have impact on the macrostructural and functional development of the brain in the minority of patients since it is well known that the onset age of BECTS is the growth period of children. Seizures are caused by abnormal neuron discharges, which can lead to brain metabolism dysfunction. The human brain is a complex network consisting of different brain regions. The structural integrity and normal function of the brain network significantly affect human cognitive function. Epilepsy is a neural network disease, and frequent discharges can lead to structural damage to the brain and changes in functional connections, thereby affecting the brain network (Galanopoulou and Moshé, 2009). There is a general similarity between the brain resting-state network (RSN) and the brain task-state network (Cheng et al., 2018). Therefore, the possible mechanism of cognitive dysfunction can be understood by observing the dynamic changes of resting-state networks (RSNs). We found in epilepsy patients that the RSNs associated with cognition and presenting significant discrepancy are usually: default mode network (DMN), central executive network (CEN), and salience network (SN) (Cataldi et al., 2013; Hu et al., 2017; Li et al., 2017; Yang et al., 2018) from studying CI in several brain network articles, which was called triple network model (Menon, 2011). The abnormal functional connectivity (FC) between and within the triple network comprises the pathogenesis of various neurological and psychiatric disorders, including Parkinson’s disease, idiopathic generalized epilepsy, depression, and anxiety disorders (Menon, 2011; Putcha et al., 2016; Wei et al., 2016). It helps us understand cognitive dysfunctions in BECTS. Moreover, these neurocognitive networks are anchored in some key nodes. The DMN mainly consists of the posterior cingulate cortex (PCC), precuneus (PCu), and medial prefrontal cortex (MPFC). The CEN primarily comprises the dorsolateral prefrontal cortex (DLPFC) and the posterior parietal cortex (PPC). The SN includes classical components such as the anterior insular cortex (AI) and anterior cingulate cortex (ACC) (Wei et al., 2016). Accordingly, the magnetic source activity in critical nodes of DNM, CEN, and SN can be altered in BECTS, underlying the cognition dysfunction in BECTS patients.

Previous studies on BECTS primarily utilized functional magnetic resonance imaging (fMRI) and EEG and indicated that brain activity exhibits frequency-dependent properties (Jacobs et al., 2008; Gohel and Biswal, 2015). Magnetoencephalography (MEG) is a non-invasive and direct technique having a high spatiotemporal resolution that can complete magnetic source imaging at a millisecond level (Wilson et al., 2016). It can quantify magnetic fields generated from brain activity not attenuated by the scalp and skull (Guggisberg et al., 2008; Burgess, 2011). Therefore, MEG has been widely used in multi-frequency brain activity analysis of children suffering from BECTS.

The Wechsler intelligence test was used to divide patients into the CI group and the cognitive non-impairment (CNI) group. The healthy group was also tested. We aimed to utilize MEG to investigate the magnetic source activity of the BECTS and the healthy controls from low - to high-frequency bands for exploring dynamic changes in neurocognitive networks, contributing to our further understanding of the mechanism of CI in patients with BECTS.

## 2. Materials and methods

### 2.1. Participants

Fifty patients diagnosed with BECTS were recruited from the Neurology Department of the Nanjing Brain Hospital and Nanjing Children’s Hospital of China. Meanwhile, we initiated socially-oriented recruitment for the healthy control group, enrolling 24 children. All the patients were diagnosed by meeting the International League Against Epilepsy (ILAE) 2017 criteria. Moreover, all the enrolled patients were included in the study without taking antiepileptic drugs (AEDs).

The inclusion criteria for patients were: (1) conformed to the classification of epilepsy syndrome using the ILAE2017; (2) not taking any AEDs; (3) aged 6–14 years; (4) normal mental and physical development, no mental or somatic disease or neurodevelopmental delay. The exclusion criteria were: (1) the presence of implanted mental devices, including artificial pacemakers, which could create obvious noise and interfere with the MEG results; (2) evidence of intellectual disability, neurological disorders, and significant systemic organ disorders; (3) history of trauma and asphyxia during childbirth; and (4) unable to cooperate with the research process.

Of the 50 patients, one had a history of obstetric injuries, and one did not cooperate with the MRI scan. Finally, 48 children were included in the study. The Medical Ethics Committee of Nanjing Medical University, Nanjing Brain Hospital, and Nanjing Children’s Hospital Medical Ethics Committee approved the research. The parents or legal guardians of the subjects provided a signed informed consent form.

### 2.2. MEG recordings

A whole-head, CTF-275 channel MEG system (VSM Medical Technology Company, Canada) was used to record MEG signals in a magnetically shielded room at the MEG Center of Nanjing Brain Hospital. All the metal items on the subjects were removed before data collection. Then, three small electromagnetic coils were attached to the nose root and bilateral pre-auricular ears of each subject, which helped measure head positions relative to the MEG sensors. During the recording, subjects were asked to remain silent and close their eyes gently. Meanwhile, the subjects had to keep their head fixed, quiet their mind, and stay awake. Magnetoencephalography data were recorded for 2 min at a sampling rate of 6,000 Hz, generating at least four continuous data files. The system allowed for head localization at an accuracy of 1 mm, and the head movement was limited to 5 mm. The dataset with head movement > 5 mm was disregarded, and the recording was restarted. Furthermore, we routinely recorded one MEG dataset inside an empty room to identify the noise created by the system and the environment.

### 2.3. MRI scan

After completing MEG data acquisition, all subjects underwent an MRI with a 3.0T scanner (Siemens, Germany). Three coils were placed in the nasion and pre-auricular of the participants to minimize MRI and MEG data. It was the same as the Magnetoencephalography positioning coils. All the anatomical landmarks digitized in the MEG study were identified using the MRI.

### 2.4. Neurocognitive assessment

The Wechsler Intelligence Scale for Children, fourth edition (WISC-IV) is used to assess the intelligence of children globally. Children underwent the WISC-IV test the same day for comprehensive evaluation. The analysis graphs of synthetic scores and sub-tests can provide an intuitive understanding of the overall cognitive function level. The WISC-IV had ten core and five supplemental subtests, synthesizing the FSIQ and four indices. Specifically, the four indices are the verbal comprehension index (VCI), evaluating language training levels, viz., the perceptual reasoning index (PRI), measuring the ability of nonverbal and fluid reasoning; the working memory index (WMI), reflecting the capability to use short-term memory for manipulating information; and the processing speed index (PSI), evaluating the attention and coordination of motor skills in children. Clinical psychologists performed all the tests. For healthy children, the average FSIQ score was 90–110. A score of 80–90 was at a low-middle level, 70–80 was the critical value of normal FSIQ, and < 70 indicated intellectual disability. Clinically, cognitive dysfunction in children with BECTS is an FSIQ score < 90. Therefore, it was chosen as the cut-off for cognitive decline, and the children with BECTS were divided into a CI and a CNI group.

### 2.5. Data preprocessing

We utilized the following strategies to exclude non-brain and environmental artifacts from spontaneous MEG data: (1) all the data were visually inspected for containing artifacts due to head movements or environmental noise, and any contaminated segments were discarded; (2) notch filters (50 Hz and its harmonics) were utilized to remove powerline contamination; (3) the MEG recordings started with a 2 min empty-room recording for obtaining environment and sensor noise. The noise covariance was calculated using the offline source analysis to take the remnant and stationary instrumental, sensor, and environmental noise components into account. We used the FreeSurfer image analysis suite^1^ to automatically reconstruct the T1-weighted structural volumetric images into the surface model for further source analysis. The software completed the detailed geometric reconstruction of the scalp, brain gray, and white matter, providing a three-dimensional topographical representation of the brain surface. Then, it was utilized to assess the gray and white matter boundaries.

We retained all the MEG recordings without prolonged artifacts. Based on the preliminary work of our team (Sun et al., 2020, 2021), we found that a 30 s time period is sufficient to ensure the stability of the data. Spike discharges disrupt resting brain activity (Fahoum et al., 2013) and have an effect on magnetic source activity. Thus, an interictal waveform that lasted the 30 s while avoiding the spikes was chosen. Then, the selected MEG data were analyzed in eight predefined frequency bands: delta (2–4 Hz), theta (5–7 Hz), alpha (8–12 Hz), beta (15–29 Hz), gamma1 (30–59 Hz), gamma2 (60–90 Hz), ripple (90–250 Hz), and fast ripple (250–500 Hz).

### 2.6. Minimum norm estimate analysis

We used depth-weighted minimum-norm estimation (MNE) to obtain the distributed source model of the MEG signals. The method is capable of detecting simultaneously distributed current sources scattered along the entire cortical surface and observing source-based dynamic current strength, offering great spatial accuracy through depth weighting. Several previous studies have demonstrated that the MNE method is stable (Kanamori et al., 2013; Li et al., 2022). Then, we used a multiple overlapping spheres model during the calculation to make a forward solution come true. This gives each current dipole the meaning of a cortical vertex and included approximately 15,000 vertices. Then, the following inverse operator was used to estimate the current source distribution: (1) the orientations of the source was confined to be normal to the cortex surface; (2) the depth-weighted algorithm made up for the inhomogeneous sensitivity with the orientation and the depth of the current flow; (3) the regularization parameter λ^2^ = 0.33 was used to achieve a minimum level of numerical instability, decrease the sensitivity of MNE to noise, and effectively attain a spatially smoothed solution, which has a definition of the reciprocal of the signal to noise ratio (SNR) of the MEG recordings. We can download Brainstorm freely online under the GUN general public license^2^ to perform the depth-weighted MNE analysis.

**FIGURE 1:**
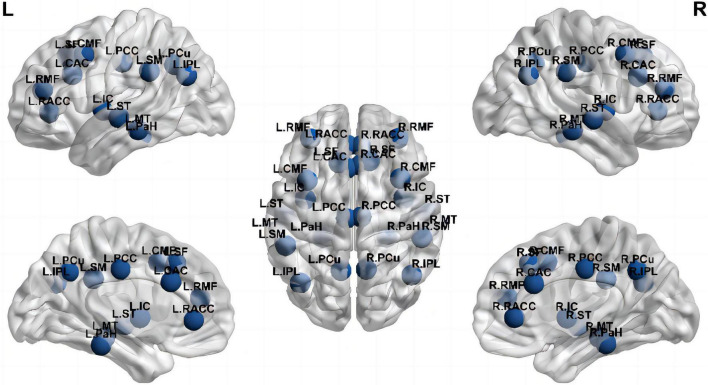
Nodes at their approximate anatomical location. Labels are the node abbreviations in the text [Generated with BrainNet Viewer (Xia et al., 2013)].

The method used to set default coordinates for anatomical fiducials on Brainstorm involved: NAS = nasion, LPA = left pre-auricular point, RPA = right pre-auricular point, AC = anterior commissure, PC = posterior commissure, and IH = interhemispheric point. Moreover, the default coordinates could be registered using the MEG coordinates. We also used Brainstorm’s *spm_maff8* function from SPM12 to register the T1 MRI for each subject to the Montreal Neurological Institute (MNI) coordinate system, automatically calculating a 4 × 4 affine transformation. We defined regions of interest (ROIs) in the T1 template volume with the Desikan-Killiany cortical parcellation. Depending on previous studies of cognitive-related brain areas (Wei et al., 2016), 26 brain areas were selected as the ROIS: bilateral caudal anterior cingulate cortex (CAC), caudal middle frontal cortex (CMF), inferior parietal lobule (IPL), insula cortex (IC), MT, parahippocampal cortex (PaH), PCC, PCu, rostral anterior cingulate cortex (RACC), rostral middle frontal cortex (RMF), superior frontal cortex (SF), ST, and SM, which are the main components of the three networks associated with cognition, namely DMN, CEN, and SN, respectively. Figure 1 showed the nodes localization. The relative current strength of all vertices in the ROI was calculated to estimate the oscillatory power based on the source. The Welch technique (window duration 5 s with 50% overlap) was utilized to determine the power spectral density (PSD) for each ROI. The PSD values represented the spectral power of each ROI and were scaled to the total power over the whole frequency spectrum at each frequency bin: Relative PSD(f) = PSD(f)/∑_i_[Total PSD(f_i_)], where *f*_*i*_ is the individual frequency from the absolute PSD. The numerator of the formula indicates the original PSD value of the current frequency band, and the denominator of the formula indicates the total of the original PSD values of all selected frequency bands. The relative PSD value represented the current frequency band’s contribution to the total signal power, which is located between 0 and 1. It has been reported that PSD values across brain regions and subjects are standardized using the procedure (Niso et al., 2019).

### 2.7. Statistical analysis

The independent-sample *t*-test was used to compare the clinical and demographic information among the three groups. The Shapiro-Wilk test was used to evaluate the normality of the data set, and the Kruskal-Wallis test was used to compare group differences in the spectral power of each ROI in different frequency bands among the three groups. Pearson or Spearman correlation analysis was used to analyze the correlation between the clinical features and the spectral power within each frequency band. The statistical significance threshold was set at *p* < 0.05. Bonferroni multiple comparisons were used for multiple comparisons. Then, typeIerrors were controlled using the false discovery rate (FDR) controlling procedure. Data analyses and computations were performed with SPSS25.0 (SPSS Inc., Chicago, IL, USA).

## 3. Results

### 3.1. Clinical information

The study recruited 48 patients with BECTS and 24 healthy controls. All the participants were subjected to neuropsychological tests by a specialist neurologist with WISC-IV. Twenty-four patients had an FSIQ < 90, and 24 had an FSIQ > 90. We defined them as the CI group and the CNI group, respectively. The mean age of the CI group was 7.98 ± 1.45 years, and the CNI group was 8.80 ± 1.74 years, and for the healthy group was 8.35 ± 1.92 years. The average epilepsy course for the CI group was 6.08 ± 4.14 months and 5.40 ± 7.75 months for the CNI group. In the CI group, the number of seizures was 3.17 ± 1.34, while it was 3.125 ± 2.95 in the CNI groups. The WISC-IV scores of BECTS with CI were: FSIQ = 84.63 ± 4.79, VCI = 82.96 ± 9.12, PRI = 88.5 ± 10.44, WMI = 88.29 ± 8.47, and PSI = 93.25 ± 15.88. The WISC-IV scores of BECTS with non-cognitive impairment included: FSIQ = 107.79 ± 8.03, VCI = 104.96 ± 12.23, PRI = 111.79 ± 7.37, WMI = 100.79 ± 10.01, and PSI = 106.16 ± 14.54. Specific details of the patient’s clinical information are shown in Table 1.

**TABLE 1:** Clinical information of patients.

Clinical characteristics	The CI group	The CNI group	*P*-value
Number	24	24	
Sex, F/M	14/10	11/13	
Age at seizure onset, y	7.98 ± 1.45	8.80 ± 1.74	0.089
The course of epilepsy, months	6.08 ± 4.14	5.40 ± 7.75	0.714
The number of seizures	3.17 ± 1.34	3.125 ± 2.95	0.951
**The WISC-IV scores**			
FSIQ	84.63 ± 4.79	107.79 ± 8.03	<0.001**
VCI	82.96 ± 9.12	104.96 ± 12.23	<0.001**
PRI	88.5 ± 10.44	111.79 ± 7.37	<0.001**
WMI	88.29 ± 8.47	100.79 ± 10.01	<0.001**
PSI	93.25 ± 15.88	106.16 ± 14.54	0.006**

CI, cognitive impairment; CNI, cognitive nonimpairment; F, female; M, male; y, years; FSIQ, full-scale intelligence quotient; VCI, verbal comprehension index; PRI, perceptual reasoning index; WMI, working memory index; PSI, processing speed index.

*p < 0.05, **p < 0.01 after Bonferroni correction for multiple comparisons.

### 3.2. Cortical activation localization

**FIGURE 2:**
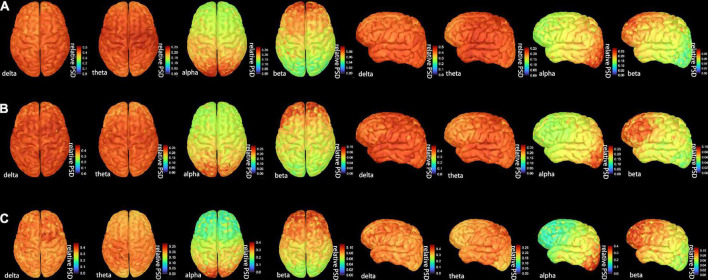
The distribution of the whole-brain average cortical activation in resting-state condition in **(A)** the cognitive impairment (CI) group, **(B)** the cognitive nonimpairment (CNI) group, and **(C)** healthy controls. We showed only four frequency bands with the most significant differences. The activation maps were shown in two views: top and dorsal; the current power of the underlying cortical sources were color-coded, as the values increased, the color gradually changed from blue to red. PSD = relative power spectral density. The complete distribution of the whole-brain average cortical activation are shown in Supplementary Figure 1.

Figure 2 shows the grand-averaged activation distributions on the cortical surfaces of bilateral hemispheres from delta to beta bands across the CI, CNI, and HC groups. There was a similar pattern of cortical activation across the three groups. Moreover, the distribution of magnetic sources indicated a uniform and widespread characteristic along the low-frequency bands (delta, theta). However, the activity of the parieto-occipital region was outstanding in the alpha band. The activation intensity of the three groups revealed differences in different frequency bands. The CI group had the strongest activation in the delta band, the CNI group possessed the strongest activation in the theta band, and the HC group indicated the most vigorous magnetic source intensity within the alpha band.

### 3.3. Spectral power density of the resting-state network

**FIGURE 3:**
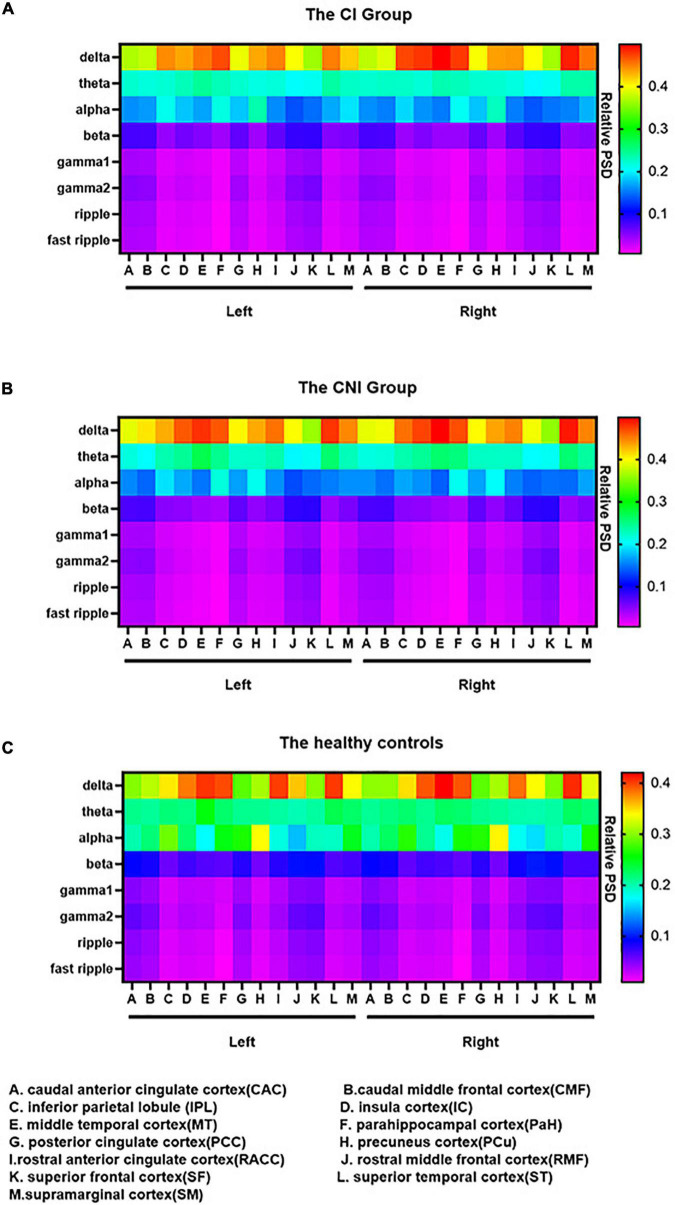
The relative spectral power of 26 brain regions from delta to fast ripple bands in **(A)** the cognitive impairment (CI) group, **(B)** the cognitive nonimpairment (CNI) group, and **(C)** healthy controls. The data were color-coded. In **(A)** the CI group and **(B)** the CNI group, delta band had a diffuse enhancement within bilateral IPL, IC, MT, PaH, PCu, RACC, ST and SM. In **(C)** the healthy controls, the alpha band had a significant diffuse enhancement, especially within bilateral PCu. PSD = relative power spectral density.

**FIGURE 4:**
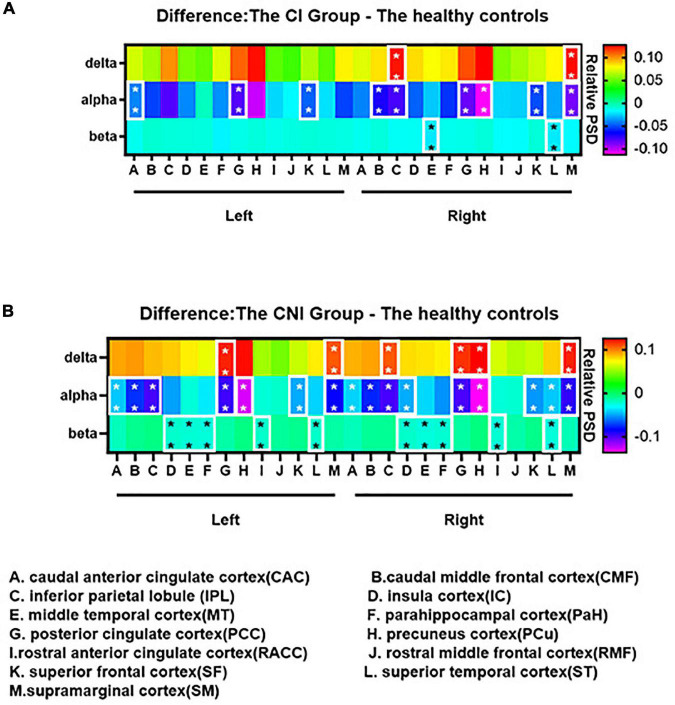
Group differences of the spectral power among the delta, alpha and beta bands: **(A)** The cognitive impairment (CI) group - the healthy controls; **(B)** the cognitive nonimpairment (CNI) group - the healthy controls. The difference maps were color-coded, as the values increased, the color shifted from purple to red. In **(A,B)**, compared to the healthy controls, the spectral power of the CI group and the CNI group significantly decreased in the alpha band. We didn’t find any differences between the CI and the CNI group in all frequency bands. *p < 0.05, **p < 0.01 after Bonferroni correction for multiple comparisons. The complete group differences of the spectral power from delta to fast ripple bands are shown in Supplementary Figure 2.

The relative spectral power of each ROI in the CI, CNI, and HC groups ranged from delta to fast ripple bands significantly differed in Figure 3. The normalized PSD of the ROIs in most frequency bands significantly altered between the CI and the CNI group. The ROIs of the CI group had the highest activation in the delta band, similar to the cortical activation distributions in Figure 2. Moreover, the ROIs of the CNI group had higher spectral power in the theta band than the other two groups. The HC group indicated the most potent activation in the alpha band. In other words, the magnetic source activation of the BECT group reduced than the HC group in the range of 8–12 Hz. The differences in magnetic source localization mainly focused on the delta, alpha, and beta bands. Figure 4 demonstrates the magnetic spectral power differences of the ROIs for each frequency band among the two groups (CI-HC and CNI-HC). Therefore, the results with *p* < 0.005 were listed in the text. The complete results are presented in the Supplementary Tables 1, 2. At the delta (2–4 Hz) band, significant differences were observed: between the CI and the HC group, SM.R: *P* = 0.001, IPL.R: *P* = 0.003; between the CNI and the HC group, SM.R: *P* < 0.001, SM.L: *P* = 0.002, PCC.L: *P* = 0.003. Significant differences were observed at the theta (5–7 Hz) band: between the CNI and the HC group, IC.R: *P* = 0.02. For the alpha (8–12 Hz) band, significant differences were found: between the CI and the HC group, CMF.R, SM.R: *P* < 0.001, PCC.R, SF.L, SF.R: *P* = 0.001, PCC.L: *P* = 0.002, IPL.R: *P* = 0.003; between the CNI and the HC group, and CMF.L, CMF.R, IPL.R, PCC.L, PCC.R, SF.L, SF.R, SM.L, SM.R: *P* < 0.001, IPL.L, PCu.R: *P* = 0.001, CAC.L: *P* = 0.002, CAC.R, PCu.L: *P* = 0.003. At the beta (15–29 Hz) band, significant differences were indicated: between the CNI and the HC group, MT.L: *P* < 0.001, IC.L, MT.R, PaH.L, ST.L, ST.R: *P* = 0.001, IC.R, RACC.L, RACC.R: *P* = 0.002, PaH.R: *P* = 0.003. At the gamma1 (30–60 Hz) band, the CNI and the HC group had significant differences, MT.L, PaH.L: *P* < 0.001, MT.R, PaH.R, ST.R: *P* = 0.001, IC.L, IC.R, ST.L: *P* = 0.002. At the gamma2 (60–90 Hz) band, significant differences could be seen: between the CNI and the HC group, MT.L, PaH.L, PaH.R: *P* = 0.001, MT.R: *P* = 0.002, IC.L, ST.R: *P* = 0.002. At the ripple (90–250 Hz) band, it was observed that: between the CNI and the HC group, PaH.R: *P* = 0.002, MT.R, PaH.L: *P* = 0.003, MT.L, ST.R: *P* = 0.004. At the fast ripple (250–500 Hz) band, significant differences were observed: between the CNI and the HC group, MT.R, ST.R: *P* = 0.002, IC.R, MT.L, PaH.R: *P* = 0.003. There was no statistically significant difference between the CI and the CNI group across all the frequency bands.

### 3.4. Correlation analysis

**FIGURE 5:**
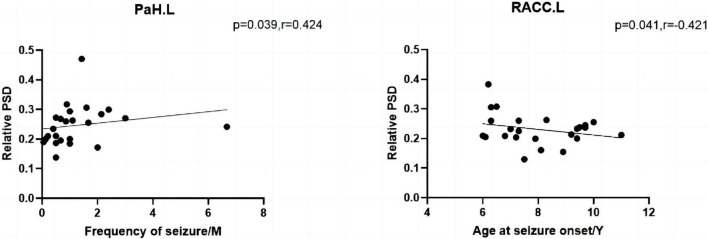
The left shows that the frequency of seizure of the CNI group was negatively associated with relative spectral power in the theta band. The right shows that the age at seizure onset of the CI group was positively associated with relative spectral power in the theta band. M, months, Y, years, PSD, relative power spectral density. *p < 0.05 after FDR multiple comparisons.

The results of the correlation analysis are depicted in Figure 5. In the CI group, age at seizure onset was negatively associated with the relative spectral power of RACC.L (*P* = 0.041, *R* = –0.421) in the theta band. The frequency of seizure in the CNI group was positively associated with the spectral power of the relative spectral power of PaH.L (*P* = 0.039, *R* = 0.424) within the theta band. All the comparisons survived FDR correction for multiple comparisons.

## 4. Discussion

This study classified children based on the presence of CI. We attempted to investigate the spectral power changes between BECTS patients with and without CI and healthy controls across different frequency bands. The brain areas were examined between three networks associated with cognition, viz., DMN, CEN, and SN. Although previous studies revealed that children with BECTS had cognitive decline, few studies investigated the pathogenesis of CI. Furthermore, previous studies focused on the changes in RSNs in patients with BECTS. Among them, MEG had been widely used. In contrast to EEG, MEG can directly measure changes in the neural magnetic signal of brain cells. The magnetic signal passes through the skull and brain tissue without signal distortion (Plummer et al., 2019). MEG allows localization of tangential dipoles and estimation of deep brain endogenous sources, whereas EEG is susceptible to volumetric effects (Cohen and Cuffin, 1983). Thus, the differences in neuromagnetic source strength among the study groups complained about the different neural activity patterns involved in seizures and CI. This is useful for understanding brain changes during the early stages of disease development.

### 4.1. Clinical characteristics and neuropsychological tests

Incecik et al. (2015) reported that seizure onset before 4 years old contributed to the AED response failure. The earlier the onset, the worse the drug response and the higher the likelihood of cognitive decline, which is consistent with our findings. We observed that the age of onset in the CI group is younger than in the CNI group. The characteristic development of the nervous system in children is “fast followed by slow.” Around 5–6 years old is the prime period of intellectual development for a child. We speculated that premature seizures affected the connections between neurons in the brain, causing cognitive decline. Besides, the average course of epilepsy in the CI group was longer than in the CNI group in the study. Like Shehata and Bateh Ael (2009) reported before, long epileptic seizures could reduce the ability to resist seizures and respond to the AEDs (Galanopoulou and Moshé, 2009), turn neurotransmitters overexcited, and damage the neural structures. Furthermore, previous studies have indicated that long-term interictal spike discharges can affect the development and function of nervous networks and impact brain function on both short and long-term levels (Ebus et al., 2012; Drane et al., 2016).

Previous studies have revealed that the FSIQ of patients with BECTS was normal (Giordani et al., 2006; Verrotti et al., 2011). We observed that the unmedicated children with BECTS scored lower on cognitive function than healthy controls. Around 50% of children with BECTS had FSIQ < 90 scores. Furthermore, BECTS patients had significantly lower scores on full-scale IQ, verbal comprehension, perceptual reasoning, working memory, and processing speed than healthy controls. Multiple studies have indicated that BECTS is associated with intellectual disability and CI, consistent with our research. The brain network is the psychological basis of the cognitive activities of the brain (McIntosh, 2000). Recurrent clinical seizures cause functional abnormalities in cognitive-related brain networks, and the pathological mechanisms require further study.

### 4.2. Cortical activation localization

The whole brain activation map was similar across the three groups. The CI group from our study had the strongest activation in the delta band, the CNI group showed the highest magnetic source intensity in the theta band, and the HC group was the strongest in the alpha band. These phenomena could be due to the spreading of epileptic discharges from the epileptogenic zone (Hsiao et al., 2015). However, the results were acquired by visual inspection and were based on the color-coded cortical activation strength, which may be inaccurate. Thus the PSD values of the cognitive-related brain networks will be elaborated.

### 4.3. Spectral power density of the resting-state network

Human well-level cognition depends on the dynamic balance of changes within brain networks, leading to cognitive dysfunction from its abnormalities. A previous study had focused on core cognitive networks and found that interactions between specific neural networks may be new clues to CI (Wei et al., 2016; Yang et al., 2018). In the present whole-head MEG study, BECTS patients showed resting-state brain activity characterized by widespread increases in delta band, a diffuse decrease in alpha, and beta power, together with a reduction in the temporal lobe, insula gyrus, and parahippocampal gyrus in gamma1, gamma2, ripple, and fast ripple bands.

DMN has been reported to be associated with CI as a baseline state of the human brain (Raichle et al., 2001). The DMN involves the PCC, precuneus cortex, parahippocampal cortex, RACC, and superior frontal and temporal cortex (Raichle et al., 2001; Chen et al., 2018). During rest, sleep, and not being engaged in an attention-demanding task, DMN is activated, and DMN is suppressed when performing a task (Raichle et al., 2001). DMN is associated with performance in various cognitive functions, such as attention control (Weissman et al., 2006), introspective-orientated thought (Gusnard et al., 2001), working memory (Esposito et al., 2006), and social behaviors (Lynch et al., 2013).

DMN is crucial in BECTS. In previous studies, the most common finding is that connectivity is decreased within many component regions of DMN. Our study indicated that the spectral power inside the bilateral ACC and PCC/precuneus region, bilateral superior and middle temporal cortex, bilateral IPL, and bilateral SM region in children with BECTS enhanced in the delta band, together with decreased spectral power in the alpha band. Furthermore, we found that the spectral power of children with BECTS decreased within the bilateral superior and middle temporal cortex, the bilateral ACC region, and the bilateral PaH and right SM region in the beta band. A similar activation pattern was observed in the gamma1, gamma2, ripple, and fast ripple bands: the spectral power decreased within the bilateral superior and middle temporal cortex, bilateral PaH, and bilateral ACC regions.

The changes in DMN mainly focus on the PCC/precuneus region and parietal cortex (Xiao et al., 2016) in children with BECTS. Besides, Li et al. (2022) found an enhancement of spectral power intensity within the PCC region in the delta band and a decrease of spectral power in the alpha band while studying the whole brain power spectrum of BECTS with mild SWI. The dominant rhythm of healthy people awake and with their eyes closed are alpha rhythm. However, patients with BECTS show an increase in slow activity and a shift of the alpha peak toward the delta rhythm, similar to previous studies (Miyauchi et al., 1991). Spectrum slowing has been associated with cognitive decline and induces clinical seizures. Bosboom et al. (2006) investigated the MEG relative spectral power of resting-state between demented and non-demented Parkinson’s disease (PD). They found brain activity characterized by widespread increases in delta and theta power, along with a diffuse decrease in alpha, beta, and gamma power, closely related to CI in PD. Furthermore, Schoonhoven et al. (2019) observed that theta power increase was strongly associated with impaired cognition. It means that the increase of slow activity could be the pathogenesis of BECTS, causing CI and a worse prognosis than patients without slowing (Miyauchi et al., 1991). A study on Alzheimer’s disease (AD) reported a significant elevation in the delta band and significantly reduced alpha power (Laptinskaya et al., 2020). Similar to our studies, the magnetic source alternation in different bands using MEG could be a cognition marker in patients with BECTS. The PCC/precuneus is a significant hub of the DMN and has the most active metabolism in the whole brain cortex (Raichle et al., 2001). PCC has a vital role in the broad and continuous sampling of external and internal environments, whose abnormality may be associated with memory dysfunction and emotional processing failure (Broyd et al., 2009). The enhancement of the PCC region in the delta band is closely related to cognitive decline in patients with BECTS. ACC region plays an important role in monitoring, control, and economic function, and its dysfunction led to an inaccurate performance in the attention control task (Broyd et al., 2009). In a previous study, the activities of cortical sources of the frontal lobe decreased in the beta band in patients with BECTS. Moreover, they found that the phenomenon may be associated with DMN suppression (Adebimpe et al., 2015). The primary role of the IPL is concentration and stimulus surveillance (Singh-Curry and Husain, 2009). Thus, the dysfunction of IPL could have contributed to the attention drop in patients with BECTS. The parahippocampal cortex is involved in the limbic system regulating inner organs, emotions, feelings, study memory, etc. The decrease of the parahippocampal cortex in the high-frequency section may indicate that epileptic discharge in BECTS caused complete suppression of the whole brain network, then led to cognitive decline in patients with BECTS. Our study could shed light on the previous neuropsychological assessments of children with BECTS who are distracted, anxious, and depressed.

The CEN is a part of the core neurocognitive networks (Wei et al., 2016) and is involved in behavior, memory, emotion, and control processes (Seeley et al., 2007). CEN primarily consists of the bilateral DLPFC, ventrolateral and dorsomedial PFC, lateral parietal cortex, and bilateral ACC region, which is responsible for maintaining working memory, problem-solving, and making a decision (Seeley et al., 2007). The dysfunction of CEN has been reported to be involved in various neuropsychiatric disorders (Seeley et al., 2007). In previous studies, researchers observed that the CEN regions indicated dynamic deactivation, leading to epileptic discharges and cognitive dysfunction (Zhang et al., 2014). The CEN depicted extensive inhibitions in higher-order cognitive processes during epileptic seizures (Zhang et al., 2014). Similarly, we found that the spectral power within bilateral CMF and bilateral ACC region in patients with BECTS enhanced in the delta band, with decreased spectral power within bilateral CMF, bilateral ACC region, right RMF, and bilateral SF region within the alpha band. Furthermore, the spectral power of children with BECTS decreased within the right SM region in the beta band. The reduced activation of the MF and the SF region has been observed in many studies of cognitive control over socially relevant tasks with and without emotion. It could shed light on the dysfunction of the CEN in the cognitive processes (Qiu et al., 2011). Fan et al. (2007) observed that the activity in beta and low gamma bands decreased in attention networks. The decrease in the high-frequency band could be due to the disruption of the CEN core neural network, directly leading to CI. DLPFC plays a vital role in neuropsychological function. Thus, we speculate that patients with BECTS could have some defects in psychological and emotional aspects. The activation of CEN and SN typically increases during processing cognitive and affective information driven by stimulus, and their response increases or decreases proportionately with the cognitive task demands (Menon, 2011). In the future, the mechanisms of CEN and SN need further investigation under task state to evaluate the cognitive function of BECTS during the early stages.

Many foci were identified as part of the SN network, anchored in the dorsal anterior cingulate cortex (dACC) and frontoinsular cortex (FIC), such as ACC, anterior insula (AI) amygdala, and thalamus (He et al., 2020). SN detects, integrates, and filters automatic, interoceptive, and emotional information (Seeley et al., 2007). The model had been put forward that the SN initiators switch networks leading to CEN engagement and DMN disengagement (Menon and Uddin, 2010). The disruption of the integrity or connectivity of the SN led to deficits in cognitive functioning. FIC plays an essential role in switching between the CEN and the DMN. Our study found that the spectral power within the bilateral ACC region enhanced in the delta band and decreased in the alpha band in patients with BECTS. Furthermore, the spectral power within the right IC enhanced in the theta band and decreased in the alpha band in patients with BECTS. Besides, the spectral power within bilateral ACC and bilateral IC region decreased in beta, gamma1 bands, gamma2, ripple, and fast ripple bands. They found that one of the changes in AD in the early stage is the increase in theta power (van der Hiele et al., 2007). He et al. (2020) observed that SN deactivation is related to the lack of concentration in patients with BECTS. Luo et al. (2014) found that the functional integration at the ACC within the SN increased and decreased at the right IC in epilepsy patients. Besides, a previous study observed a disconnection between the ACC and the IC in epilepsy patients (Killory et al., 2011). Epileptic discharges interfered with the connections within SN and resulted in the deficit of processing salient information. Furthermore, the magnetic source inactivation and the changes in a functional network connection could be interconnected. The inactivation of the SN leads to changes in the functional network connection. Thus, it impairs the cognitive function of patients with BECTS. The spectral power of the CI group and the CNI group indicated obvious significant compared to the healthy controls, respectively. However, We didn’t find any statistically significant difference between the CI and the CNI group across all the frequency bands. This may signify that the BECT group showed CI compared the HC group in early stage. Although the FSIQ of the BECTS group were normal, the neuromagnetic activity had changed distinctly. We assume the result emphasizes the importance of early treatment.

Therefore, the increase in slow activity indicated clinical seizures and led to subtle cognition changes. The enhanced spectral power intensity in the delta and theta band could be an imaging marker suggesting early seizure. To the best of our knowledge, this is the first time we have systematically studied the neurocognitive core network changes at the magnetic source spectrum power level. It has been reported that high-frequency magnetic source signals in epilepsy patients are highly localized within the epileptogenic focus (Xiang et al., 2009). Alegre et al. (2006) reported that high-frequency epileptic activity could have a causal role in seizures. The changes in the high-frequency spectrum in our study are consistent: the spectral power density within IC, ST, MT, and PaH decreased. The study of high-frequency activity could lead to a new understanding of the seizure mechanisms of BECTS.

### 4.4. Correlation analysis

This study found that the frequency of seizures was positively associated with the spectral power of the left PaH. We also observed a negative association between the age of seizure onset and the spectral power for the left RACC. Early onset age and more frequent seizures are risk factors for poor prognosis (Incecik et al., 2015). Frequent discharges destroy neurons, disrupt functional brain networks and cause CI in patients with BECTS. Epileptic activity recedes with increasing age, consistent with the self-limiting definition of BECTS. Incecik et al. (2015) found that an earlier age at the onset of seizure causes a worse response to AEDs. It suggests that the CI in patients with early-onset epilepsy is more severe. Although BECTS is considered benign, abundant studies have reported hat patients with BECTS have multiple neuropsychological impairments. This could suggest the importance of treatment during the early stage.

### 4.5. Limitations

However, there are some limitations to our study. First, the sample size of our study is small. Therefore, expanding the number of participants could help us discover more significant differences between the groups. Second, we used only the WISC-IV to evaluate the cognitive function of the patients. However, it cannot systematically assess cognition like language function. Third, although we minimized the artifacts, the artifacts and noise with the MEG signal could not be eliminated completely. Finally, the FC between and within brain regions was not included in evaluating dynamic changes within cognitive networks. Therefore, we plan to explore the alternation of the FC within neurocognitive core networks in BECTS in future studies to investigate its physiopathologic mechanism.

## 5. Conclusion

Our study demonstrated that DMN, CEN, and SN brain regions among patients with BECTS present characteristic alternations in the level of spectral power. The spectral power varies with different frequency bands. Patients in the CI and CNI groups depicted a similar magnetic source activity pattern. Unlike adult epilepsy, we could not find any difference between the CI and the CNI group. Children with BECTS having CI indicated an earlier age at seizure onset, and we found higher seizure frequency in children with BECTS without CI. Furthermore, BECTS have a diffuse impairment in cognitive function. The spectral power of brain regions within DMN, CEN, and SN in the delta and alpha bands may be a useful imaging marker for diagnosing early BECTS. The changes in the high-frequency spectrum can decipher the seizure mechanisms of BECTS.

## Data availability statement

The raw data supporting the conclusions of this article will be made available by the authors, without undue reservation.

## Ethics statement

This studies involving human participants were reviewed and approved by Medical Ethics Committee of The Affiliated Brain Hospital of Nanjing Medical University and The Affiliated Children’s Hospital of Nanjing Medical University in China. Written informed consent to participate in this study was provided by the participants’ legal guardian/next of kin.

## Author contributions

SW, YW, YHL, and FS designed the study. SW, PW, KN, YX, and YZL acquired the raw data. SW, YW, JS, and QC analyzed the data. SW wrote the manuscript. XW revised the manuscript. All authors contributed to manuscript revision, read, and approved the submitted version.

## Footnotes

1
http://surfer.nmr.mgh.harvard.edu

2
http://neuroimage.usc.edu/brainstorm
