# Supplementary material for: Alternations of neuromagnetic activity across neurocognitive core networks among benign childhood epilepsy with centrotemporal spikes: A multi-frequency MEG study
Source: Front Neurosci. 2023 Feb 22;17:1101127. doi: 10.3389/fnins.2023.1101127 (PMC9992197; doi:10.3389/fnins.2023.1101127)
Supplement: Supplementary file 1 [file Table_1.DOCX]

**Supplementary material of section 3.3**

**The D-value table for the magnetic spectral power differences of the ROIs for each frequency band among the three groups (CI-HC, CNI-HC and CI-CNI)**

**A**

**D-value: The CI Group - The healthy controls**

| **Left** | | | | | | | | | | | | | |
| --- | --- | --- | --- | --- | --- | --- | --- | --- | --- | --- | --- | --- | --- |
|  | **A** | **B** | **C** | **D** | **E** | **F** | **G** | **H** | **I** | **J** | **K** | **L** | **M** |
| **delta** | 11.458 | 10.521 | 15.917 | 8.271 | 7.917 | - | 15.625 | 15.625 | - | - | - | 9.000 | 14.292 |
| **theta** | - | - | - | - | - | - | - | - | - | - | - | - | - |
| **alpha** | -18.021 | -16.583 | -17.500 | -11.333 | - | - | -20.250 | -17.042 | - | -12.146 | -22.208 | -8.583 | -15.646 |
| **beta** | -13.417 | - | - | -11.062 | -12.271 | -14.771 | - | - | -12.688 | - | - | -12.979 | - |
| **gamma1** | - | - | - | -11.604 | -13.979 | -15.917 | - | - | -11.375 | - | - | -12.792 | - |
| **gamma2** | - | - | - | -10.438 | -11.917 | -14.604 | - | - | -10.917 | - | - | -11.271 | - |
| **ripple** | - | - | - | -8.708 | -10.729 | -13.375 | - | - | -9.708 | - | - | -10.708 | - |
| **fast ripple** | - | - | - | -8.958 | -10.938 | -10.625 | - | - | -9.792 | - | - | -10.688 | - |

| **Right** | | | | | | | | | | | | | |
| --- | --- | --- | --- | --- | --- | --- | --- | --- | --- | --- | --- | --- | --- |
|  | **A** | **B** | **C** | **D** | **E** | **F** | **G** | **H** | **I** | **J** | **K** | **L** | **M** |
| **delta** | 12.667 | 13.458 | 19.917 | 14.021 | 15.083 | - | 16.125 | 17.750 | - | - | - | 15.125 | 22.562 |
| **theta** | - | - | - | 4.625 | - | - | - | - | - | - | - | 7.021 | - |
| **alpha** | -17.250 | -22.958 | -19.750 | -15.125 | -13.208 | - | -21.375 | -19.083 | - | -17.500 | -21.833 | -15.250 | -23.125 |
| **beta** | -12.312 | - | -17.812 | -16.062 | -17.938 | -10.854 | - | - | -14.083 | - | - | -18.479 | -16.292 |
| **gamma1** | - | - | - | -15.896 | -17.375 | -13.771 | - | - | -11.750 | - | - | -17.250 | - |
| **gamma2** | - | - | - | -16.000 | -16.917 | -14.958 | - | - | -11.896 | - | - | -16.625 | - |
| **ripple** | - | - | - | -15.438 | -15.562 | -13.875 | - | - | -10.458 | - | - | -15.750 | - |
| **fast ripple** | - | - | - | -15.708 | -16.438 | -12.625 | - | - | -10.708 | - | - | -16.583 | - |

**B**

**D-value: The CNI Group - The healthy controls**

| **Left** | | | | | | | | | | | | | |
| --- | --- | --- | --- | --- | --- | --- | --- | --- | --- | --- | --- | --- | --- |
|  | **A** | **B** | **C** | **D** | **E** | **F** | **G** | **H** | **I** | **J** | **K** | **L** | **M** |
| **delta** | 17.042 | 16.792 | 16.583 | 15.292 | 16.958 | - | 19.875 | 17.000 | - | - | - | 17.250 | 20.458 |
| **theta** | - | - | - | - | - | - | - | - | - | - | - | - | - |
| **alpha** | -20.854 | -22.792 | -21.750 | -16.917 | - | - | -24.125 | -20.083 | - | -16.042 | -23.979 | -16.979 | -25.792 |
| **beta** | -17.458 | - | - | -22.250 | -23.667 | -21.854 | - | - | -20.188 | - | - | -22.146 | - |
| **gamma1** | - | - | - | -20.708 | -23.083 | -23.271 | - | - | -17.438 | - | - | -20.333 | - |
| **gamma2** | - | - | - | -19.875 | -21.083 | -21.396 | - | - | -15.271 | - | - | -18.292 | - |
| **ripple** | - | - | - | -18.667 | -19.208 | -19.688 | - | - | -15.104 | - | - | -17.604 | - |
| **fast ripple** | - | - | - | -19.104 | -19.688 | -17.562 | - | - | -14.833 | - | - | -17.812 | - |

| **Right** | | | | | | | | | | | | | |
| --- | --- | --- | --- | --- | --- | --- | --- | --- | --- | --- | --- | --- | --- |
|  | **A** | **B** | **C** | **D** | **E** | **F** | **G** | **H** | **I** | **J** | **K** | **L** | **M** |
| **delta** | 16.146 | 15.542 | 19.083 | 14.479 | 15.854 | - | 18.625 | 18.750 | - | - | - | 16.750 | 23.250 |
| **theta** | - | - | - | 16.375 | - | - | - | - | - | - | - | 15.417 | - |
| **alpha** | -19.625 | -26.729 | -23.000 | -17.875 | -16.729 | - | -24.562 | -22.167 | - | -16.375 | -24.917 | -19.000 | -25.500 |
| **beta** | -15.688 | - | -13.188 | -21.000 | -21.625 | -20.083 | - | - | -20.604 | - | - | -21.708 | -16.833 |
| **gamma1** | - | - | - | -20.354 | -21.250 | -21.292 | - | - | -16.562 | - | - | -21.062 | - |
| **gamma2** | - | - | - | -19.000 | -20.646 | -21.167 | - | - | -15.604 | - | - | -19.812 | - |
| **ripple** | - | - | - | -18.625 | -19.812 | -20.438 | - | - | -15.167 | - | - | -19.250 | - |
| **fast ripple** | - | - | - | -19.729 | -20.562 | -19.750 | - | - | -15.417 | - | - | -20.542 | - |

**C**

**D-value: The CI Group - The CNI Group**

| **Left** | | | | | | | | | | | | | |
| --- | --- | --- | --- | --- | --- | --- | --- | --- | --- | --- | --- | --- | --- |
|  | **A** | **B** | **C** | **D** | **E** | **F** | **G** | **H** | **I** | **J** | **K** | **L** | **M** |
| **delta** | -5.583 | -6.271 | -0.667 | -7.021 | -9.042 | - | -4.250 | -1.375 | - | - | - | 17.250 | -6.167 |
| **theta** | - | - | - | - | - | - | - | - | - | - | - | - | - |
| **alpha** | 2.833 | 6.208 | 4.250 | 5.583 | - | - | 3.875 | 3.042 | - | 3.896 | 1.771 | 8.396 | 10.146 |
| **beta** | 4.042 | - | - | 11.188 | 11.396 | 7.083 | - | - | 7.500 | - | - | 9.167 | - |
| **gamma1** | - | - | - | 9.104 | 9.104 | 7.354 | - | - | 6.062 | - | - | 7.542 | - |
| **gamma2** | - | - | - | 9.438 | 9.167 | 6.792 | - | - | 4.354 | - | - | 7.021 | - |
| **ripple** | - | - | - | 9.958 | 8.479 | 6.312 | - | - | 5.396 | - | - | 6.896 | - |
| **fast ripple** | - | - | - | 10.146 | 8.750 | 6.938 | - | - | 5.042 | - | - | 7.125 | - |

| **Right** | | | | | | | | | | | | | |
| --- | --- | --- | --- | --- | --- | --- | --- | --- | --- | --- | --- | --- | --- |
|  | **A** | **B** | **C** | **D** | **E** | **F** | **G** | **H** | **I** | **J** | **K** | **L** | **M** |
| **delta** | -3.479 | -2.083 | 0.833 | -0.458 | -0.771 | - | -2.500 | -1.000 | - | - | - | 16.750 | -0.688 |
| **theta** | - | - | - | -11.750 | - | - | - | - | - | - | - | 0.494 | - |
| **alpha** | 2.375 | 3.771 | 3.250 | 2.750 | 3.521 | - | 3.188 | 3.083 | - | -1.125 | 3.083 | 3.750 | 2.375 |
| **beta** | 3.375 | - | -4.625 | 4.938 | 3.688 | 9.229 | - | - | 6.521 | - | - | 3.229 | 0.542 |
| **gamma1** | - | - | - | 4.458 | 3.875 | 7.521 | - | - | 4.812 | - | - | 3.812 | - |
| **gamma2** | - | - | - | 3.000 | 3.729 | 6.208 | - | - | 3.708 | - | - | 3.188 | - |
| **ripple** | - | - | - | 3.188 | 4.250 | 6.562 | - | - | 4.708 | - | - | 3.500 | - |
| **fast ripple** | - | - | - | 4.021 | 4.125 | 7.125 | - | - | 4.708 | - | - | 3.958 | - |

- represents that multiple comparisons were not performed because the overall test did not show significant differences between samples.
