# Supplementary material for: Alternations of neuromagnetic activity across neurocognitive core networks among benign childhood epilepsy with centrotemporal spikes: A multi-frequency MEG study
Source: Front Neurosci. 2023 Feb 22;17:1101127. doi: 10.3389/fnins.2023.1101127 (PMC9992197; doi:10.3389/fnins.2023.1101127)
Supplement: Supplementary file 2 [file Table_2.DOCX]

**Supplementary material of section 3.3**

**The P-value table for the magnetic spectral power differences of the ROIs for each frequency band among the three groups (CI-HC, CNI-HC and CI-CNI)**

**A**

**P-value: The CI Group - The healthy controls**

| **Left** | | | | | | | | | | | | | |
| --- | --- | --- | --- | --- | --- | --- | --- | --- | --- | --- | --- | --- | --- |
|  | **A** | **B** | **C** | **D** | **E** | **F** | **G** | **H** | **I** | **J** | **K** | **L** | **M** |
| **delta** | 0.174 | 0.245 | 0.025* | 0.513 | 0.57 | - | 0.029* | 0.029* | - | - | - | 0.409 | 0.054 |
| **theta** | - | - | - | - | - | - | - | - | - | - | - | - | - |
| **alpha** | 0.009** | 0.018* | 0.011* | 0.182 | - | - | 0.002** | 0.014* | - | 0.133 | 0.001** | 0.466 | 0.029* |
| **beta** | 0.079 | - | - | 0.201 | 0.127 | 0.043* | - | - | 0.107 | - | - | 0.095 | - |
| **gamma1** | - | - | - | 0.164 | 0.062 | 0.025* | - | - | 0.179 | - | - | 0.103 | - |
| **gamma2** | - | - | - | 0.252 | 0.146 | 0.047* | - | - | 0.212 | - | - | 0.186 | - |
| **ripple** | - | - | - | 0.448 | 0.227 | 0.08 | - | - | 0.324 | - | - | 0.229 | - |
| **fast ripple** | - | - | - | 0.414 | 0.211 | 0.236 | - | - | 0.315 | - | - | 0.231 | - |

| **Right** | | | | | | | | | | | | | |
| --- | --- | --- | --- | --- | --- | --- | --- | --- | --- | --- | --- | --- | --- |
|  | **A** | **B** | **C** | **D** | **E** | **F** | **G** | **H** | **I** | **J** | **K** | **L** | **M** |
| **delta** | 0.108 | 0.078 | 0.003** | 0.061 | 0.038* | - | 0.023* | 0.01* | - | - | - | 0.037 | 0.001** |
| **theta** | - | - | - | 1.000 | - | - | - | - | - | - | - | 0.736 | - |
| **alpha** | 0.013* | 0.000** | 0.003** | 0.037* | 0.086 | - | 0.001** | 0.005** | - | 0.011* | 0.001** | 0.035* | 0.000** |
| **beta** | 0.125 | - | 0.01* | 0.024* | 0.009** | 0.217 | - | - | 0.059 | - | - | 0.007** | 0.021* |
| **gamma1** | - | - | - | 0.026* | 0.012* | 0.068 | - | - | 0.155 | - | - | 0.013* | - |
| **gamma2** | - | - | - | 0.024* | 0.015* | 0.04* | - | - | 0.147 | - | - | 0.018* | - |
| **ripple** | - | - | - | 0.032* | 0.03* | 0.065 | - | - | 0.25 | - | - | 0.027* | - |
| **fast ripple** | - | - | - | 0.028* | 0.02* | 0.110 | - | - | 0.229 | - | - | 0.018* | - |

**B**

**P-value: The CNI Group - The healthy controls**

| **Left** | | | | | | | | | | | | | |
| --- | --- | --- | --- | --- | --- | --- | --- | --- | --- | --- | --- | --- | --- |
|  | **A** | **B** | **C** | **D** | **E** | **F** | **G** | **H** | **I** | **J** | **K** | **L** | **M** |
| **delta** | 0.014* | 0.016* | 0.018* | 0.034* | 0.015* | - | 0.003** | 0.015* | - | - | - | 0.013* | 0.002** |
| **theta** | - | - | - | - | - | - | - | - | - | - | - | - | - |
| **alpha** | 0.002** | 0.000** | 0.001** | 0.015* | - | - | 0.000** | 0.003** | - | 0.024* | 0.000** | 0.015* | 0.000** |
| **beta** | 0.012* | - | - | 0.001** | 0.000** | 0.001** | - | - | 0.002** | - | - | 0.001** | - |
| **gamma1** | - | - | - | 0.002** | 0.000** | 0.000** | - | - | 0.012* | - | - | 0.002** | - |
| **gamma2** | - | - | - | 0.003** | 0.001** | 0.001** | - | - | 0.034* | - | - | 0.007** | - |
| **ripple** | - | - | - | 0.006** | 0.004** | 0.003** | - | - | 0.037* | - | - | 0.011* | - |
| **fast ripple** | - | - | - | 0.005** | 0.003** | 0.011* | - | - | 0.042* | - | - | 0.01* | - |

| **Right** | | | | | | | | | | | | | |
| --- | --- | --- | --- | --- | --- | --- | --- | --- | --- | --- | --- | --- | --- |
|  | **A** | **B** | **C** | **D** | **E** | **F** | **G** | **H** | **I** | **J** | **K** | **L** | **M** |
| **delta** | 0.023* | 0.03* | 0.005** | 0.05 | 0.026* | - | 0.006** | 0.006** | - | - | - | 0.017* | 0.000** |
| **theta** | - | - | - | 0.02* | - | - | - | - | - | - | - | 0.032* | - |
| **alpha** | 0.003** | 0.000** | 0.000** | 0.009** | 0.017* | - | 0.000** | 0.001** | - | 0.02* | 0.000** | 0.005** | 0.000** |
| **beta** | 0.028* | - | 0.087 | 0.002** | 0.001** | 0.003** | - | - | 0.002** | - | - | 0.001** | 0.016* |
| **gamma1** | - | - | - | 0.002** | 0.001** | 0.001** | - | - | 0.018* | - | - | 0.001** | - |
| **gamma2** | - | - | - | 0.005** | 0.002** | 0.001** | - | - | 0.029* | - | - | 0.003** | - |
| **ripple** | - | - | - | 0.006** | 0.003** | 0.002** | - | - | 0.036* | - | - | 0.004** | - |
| **fast ripple** | - | - | - | 0.003** | 0.002** | 0.003** | - | - | 0.032* | - | - | 0.002** | - |

**C**

**P-value: The CI Group - The CNI Group**

| **Left** | | | | | | | | | | | | | |
| --- | --- | --- | --- | --- | --- | --- | --- | --- | --- | --- | --- | --- | --- |
|  | **A** | **B** | **C** | **D** | **E** | **F** | **G** | **H** | **I** | **J** | **K** | **L** | **M** |
| **delta** | 1.000 | 0.898 | 1.000 | 0.736 | 0.404 | - | 1.000 | 1.000 | - | - | - | 0.516 | 0.922 |
| **theta** | - | - | - | - | - | - | - | - | - | - | - | - | - |
| **alpha** | 1.000 | 0.912 | 1.000 | 1.000 | - | - | 1.000 | 1.000 | - | 1.000 | 1.000 | 0.494 | 0.279 |
| **beta** | 1.000 | - | - | 0.192 | 0.178 | 0.723 | - | - | 0.643 | - | - | 0.388 | - |
| **gamma1** | - | - | - | 0.395 | 0.395 | 0.67 | - | - | 0.947 | - | - | 0.636 | - |
| **gamma2** | - | - | - | 0.355 | 0.388 | 0.783 | - | - | 1.000 | - | - | 0.736 | - |
| **ripple** | - | - | - | 0.298 | 0.481 | 0.888 | - | - | 1.000 | - | - | 0.761 | - |
| **fast ripple** | - | - | - | 0.279 | 0.443 | 0.752 | - | - | 1.000 | - | - | 0.715 | - |

| **Right** | | | | | | | | | | | | | |
| --- | --- | --- | --- | --- | --- | --- | --- | --- | --- | --- | --- | --- | --- |
|  | **A** | **B** | **C** | **D** | **E** | **F** | **G** | **H** | **I** | **J** | **K** | **L** | **M** |
| **delta** | 1.000 | 1.000 | 1.000 | 1.000 | 1.000 | - | 1.000 | 1.000 | - | - | - | 1.000 | 1.000 |
| **theta** | - | - | - | 0.155 | - | - | - | - | - | - | - | 0.494 | - |
| **alpha** | 1.000 | 1.000 | 1.000 | 1.000 | 1.000 | - | 1.000 | 1.000 | - | 1.000 | 1.000 | 1.000 | 1.000 |
| **beta** | 1.000 | - | 1.000 | 1.000 | 1.000 | 0.38 | - | - | 0.841 | - | - | 1.000 | 1.000 |
| **gamma1** | - | - | - | 1.000 | 1.000 | 0.639 | - | - | 1.000 | - | - | 1.000 | - |
| **gamma2** | - | - | - | 1.000 | 1.000 | 0.912 | - | - | 1.000 | - | - | 1.000 | - |
| **ripple** | - | - | - | 1.000 | 1.000 | 0.832 | - | - | 1.000 | - | - | 1.000 | - |
| **fast ripple** | - | - | - | 1.000 | 1.000 | 0.715 | - | - | 1.000 | - | - | 1.000 | - |

- represents that multiple comparisons were not performed because the overall test did not show significant differences between samples. *p < 0.05, **p < 0.01 after Bonferroni correction for multiple comparisons.
