# Supplementary figures and images for: Alternations of neuromagnetic activity across neurocognitive core networks among benign childhood epilepsy with centrotemporal spikes: A multi-frequency MEG study
Source: Front Neurosci. 2023 Feb 22;17:1101127. doi: 10.3389/fnins.2023.1101127 (PMC9992197; doi:10.3389/fnins.2023.1101127)

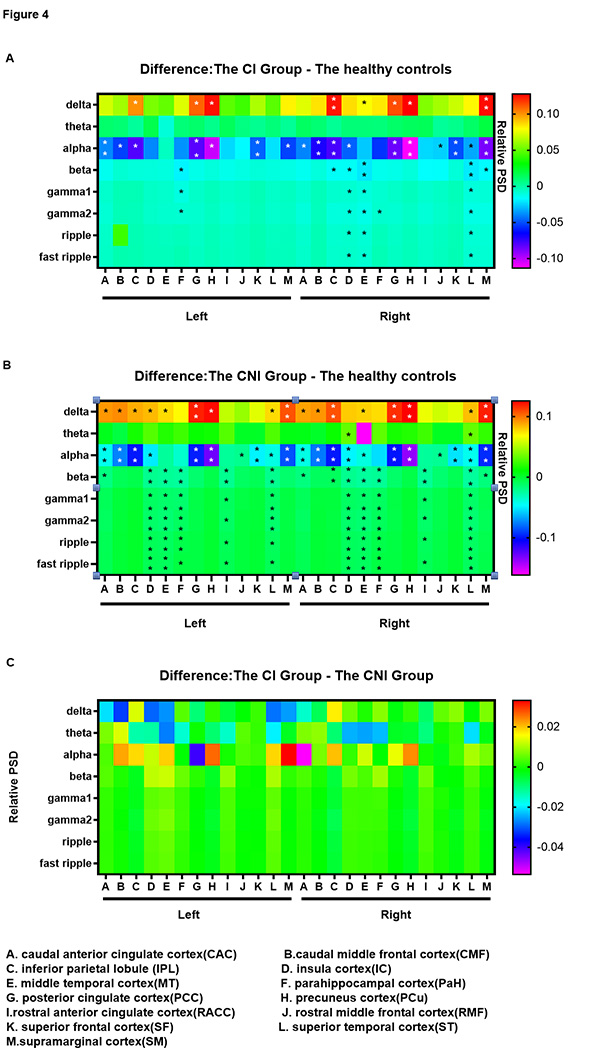

Supplement: Supplementary file 4 [file Image_2.JPEG]
